# Transformative public health education - are we there, yet?

**DOI:** 10.3389/phrs.2026.1609517

**Published:** 2026-06-15

**Authors:** Mark Avery, Suzanne Babich, Katarzyna Czabanowska, Laura Magaña, Mindaugas Stankunas

**Affiliations:** 1 Health Services Management, Department of Management, Griffith Business School, Griffith University, Nathan, QLD, Australia; 2 Indiana University Richard M. Fairbanks School of Public Health, Indianapolis, IN, United States; 3 Department of International Health, Care and Public Health Research Institute (CAPHRI), WHO Collaborating Centre for Public Health Leadership and Workforce Development, Maastricht University, Maastricht, Netherlands; 4 Department of Health Policy Management, Institute of Public Health, Jagiellonian University, Krakow, Poland; 5 Association of Schools and Programs of Public Health (ASPPH), Washington, DC, United States; 6 Department of Health Management, Lithuanian University of Health Sciences, Kaunas, Lithuania

**Keywords:** educational innovation, health systems, inclusion, public health, transformative education

## Introduction

The rapid evolution of public health challenges, including pandemics, climate change, and health inequities, requires a workforce that can adapt swiftly and continuously, along with a public health education system capable of training competent professionals.

The Special Issue of Public Health Reviews on *Transformative Public Health Education* [1] features 19 articles that inform public health educators and researchers about critical areas and trends in academic programming. The three main themes are: *Public health education and workforce development, Transformative and Inclusive approaches, and Innovation and policy in public health systems*.

### Public health education and workforce development

Traditional educational models focus on early-career training; they must change to foster lifelong learning, ensuring that professionals remain equipped with the skills and knowledge necessary to address emerging challenges.

Transformative Public Health Education highlights several innovative strategies that integrate lifelong learning into workforce development. For example, virtual interactive training courses provide timely, flexible learning opportunities. Digital platforms ensure continuous engagement with new research, policies, and best practices [2].

Other learning approaches, such as flipped classrooms and problem-based learning, foster critical thinking and problem-solving skills. In European public health programs, these methods empower ownership of education, instilling habits of inquiry that extend beyond the classroom and into careers. This approach encourages a mindset of continuous improvement and knowledge acquisition.

Programs such as the Humphrey Fellowship further strengthen the link between education and practice, offering structured career pathways that combine academic training with mentorship and leadership development, underscoring the importance of ongoing professional growth and the integration of hands-on experience.

Lifelong learning, as defined by the European Commission and UNESCO, extends beyond formal education. It encompasses a range of intentional learning activities conducted throughout life to enhance knowledge, skills, and competencies. Lifelong learning involves both formal and informal learning in diverse settings, including workplaces, homes, and communities. It plays a key role in fostering an adaptable workforce capable of responding to dynamic public health challenges.

### Transformative and inclusive approaches in public health

Public health education requires transformative and inclusive approaches that prioritize local contexts and engage diverse communities. A “grounded textual” framework blends grounded and contextual practices, focusing on regional needs while addressing universal health challenges with a deeper, more inclusive involvement of local populations in public health education, research, and policymaking. This approach goes beyond solely quantitative assessments to incorporate the rich social, cultural, and political contexts that influence health outcomes Nayar et al.

A significant example of this is the increasing involvement of people living with HIV/AIDS (PLWHA) in educational settings. PLWHA are now active contributors to public health curricula and interventions, fostering empathy, reducing stigma, and creating a more inclusive healthcare environment. Their lived experiences enrich public health education, ensuring its relevance and authenticity Namer et al.

Similarly, the World Federation of Public Health Associations (WFPHA) advocates for gender inclusivity in leadership. Despite comprising the majority of the healthcare workforce, women remain underrepresented in leadership roles. Addressing this disparity through mentorship and transparent recruitment can improve decision-making and policy development Abdalla et al.

The involvement of community health workers (CHWs) in both developed and developing countries illustrates the transformative potential of localized healthcare. CHWs bridge the gap between communities and healthcare systems, especially in maternal and child health, ensuring that vulnerable populations receive essential care Gupta and Khan.

Social prescribing has emerged as a transformative strategy in higher education. Centralized platforms, stigma-free services, and peer support are essential for engaging students in mental health and wellbeing initiatives. In this context, trained link workers or navigators, equipped with broad skills and local knowledge, are crucial Davies et al.

Finally, patient involvement in mental healthcare education is essential for developing inclusive and effective training programs. Engaging patients in educational processes takes a collaborative and holistic approach that highlights the importance of empathy and context-specific strategies [3].

These examples underscore the importance of fostering inclusion and involvement in public health, where diverse perspectives help shape the future of public health education globally.

### Transformative Public Health Education: The value and impact of innovation for health systems

Innovative policies and public health systems provide significant tangible opportunities to enhance efficiency, equity, and health outcomes [4]. These achievements increase accessibility, improve resilience, and lower costs to foster sustainable solutions to public health challenges. Innovation in policymaking promotes and enables evidence-based decision-making, which, in turn, supports new ways of working and thinking sustainably at scale. Innovation also provides an opportunity to strengthen defenses and foster resilience against emerging health threats.

Effective policies create an enabling environment while safeguarding patient and client safety and public health. The translation of innovation into practice provides the opportunity to prioritize the reduction of health disparities and enhance access for vulnerable populations. Including stakeholders is a critical aspect of innovation and promotes effective collaboration between healthcare professionals, communities, public health organizations, and government agencies.

Technological advances affect many aspects of international health, aged and social care systems, supporting innovative ideas and practices such as telehealth, which improves healthcare accessibility and efficiency [5].

Investment and expenditure for healthcare sustainability are critical policy issues and are key to enabling cost-effective, efficient opportunities for the delivery of healthcare.

Another important issue is the dynamic of resistance to change [6]. Interoperability of processes is a critical mechanism for mitigating resistance to change. Technological, procedural, or policy-driven innovations are designed to be interoperable and reduce disruptions. This drives a culture of continuous improvement while maintaining operational stability.

## Conclusion

Transformation in public health requires embedding lifelong learning into education and training systems to cultivate a workforce that is prepared and actively shaping the future of public health. Inclusivity, empathy, and context-specific strategies that prioritize the wellbeing of all communities are vital. Applying policies, innovations, technologies, and approaches to ensure quality healthcare is crucial to building a transformative workforce.

It is our sincere hope that schools and programs of public health will reflect on these trends and embrace the relevant recommendations.
